# The complete chloroplast genome of *Nymphaea thermarum* (Nymphaeaceae) from Rwanda, Africa

**DOI:** 10.1080/23802359.2021.1918030

**Published:** 2022-01-27

**Authors:** Maolin Lei, Yiheng Hu

**Affiliations:** a College of Life Sciences, Northwest University, Xi’an, China; b Institute of Botany, Chinese Academy of Sciences, Beijing, China; c University of Chinese Academy of Sciences, Beijing, China

**Keywords:** Chloroplast genome, *Nymphaea thermarum*, Phylogenetic tree, Water lily

## Abstract

*Nymphaea thermarum* is classified in the Nymphaeaceae, and is the smallest water lily in the world. It has been extinct its native environment and needs urgent protection. Here, we report and characterize the complete chloroplast genome of *N. thermarum*. The total length of the chloroplast genome is 159,849 bp and the GC content is 39.2% (A: 30.1%, C: 20.0%, G: 19.2%, T: 30.8%). The chloroplast genome consists of 8 rRNA, 37 tRNA, and 85 protein-coding genes. Phylogenetic analysis of *N. thermarum* fully resolved this taxon in a clade with *Nymphaea capensis*. The chloroplast genome of *N. thermarum* provides scientific guidance for its conservation genetics and also contributes genome resources for the phylogenetic relationship of *Nymphaea*.

Water lilies are aquatic ornamental plants of cultural and economic importance (Chen et al. 2017). They are an important horticultural plant with beautiful flowers and unique fragrances (Zhang et al., 2020). One of these species, *Nymphaea thermarum*, classified in the Nymphaeaceae, is endemic to one location, Mashyuza in southwest Rwanda (Fischer and Rodriguez 2010). It was discovered in 1987 by Eberhard Fischer (Fischer and Rodriguez 2010). It is the smallest water lily in the world with a leaf length of about 1 cm (Fischer and Rodriguez 2010; Povilus et al. 2020). Due to the destruction of the habitat caused by human activities, it has been extinct in the wild, and only part of it is cultivated. It has been listed as extinct in the wild (EW) by the International Union for Conservation of Nature (IUCN) red list (Fischer et al. 2019). Using data mining methods, here we report the complete chloroplast genome sequence of *N. thermarum* from Povilus et al. (2020), to provide a genetic basis for its conservation and phylogenetic research, and to enrich the genome resources for the Nymphaeaceae.

The *N. thermarum* specimen was collected from Rwanda (02°34′99.8′′S, 29°00′90.8′′ E) and the samples were deposited at the greenhouse of in the Arnold Arboretum at Harvard University under accession No. Rp0033 (Povilus et al. 2020, William E. Friedman, ned@oeb.harvard.edu). The raw Illumina short reads *N. thermarum* are deposited in the Sequence Read Archive (SRA) under accession number SRR8492137 (Povilus et al. 2020). The raw reads were decompressed into fastq data format using the SRA-toolkit v2.9.4 (https://github.com/ncbi/sra-tools), and low-quality bases and adapter sequences were trimmed using Trimmomatic v0.38 (Bolger et al. 2014) with default parameter settings. The filtered reads were aligned to the *Nymphaea lotus* (NC_041238) chloroplast genome using Bowtie2 v2.3.4.1 (Langmead and Salzberg 2012) with default parameters and only mapped reads were retained. The mapped reads were sorted, and duplicated reads were removed using SAMtools v1.7 (Li et al. 2009). The chloroplast genome reads were assembled using SPAdes v3.11.1 (Bankevich et al. 2012) with default parameters and yielded three large contigs with lengths of 89,999 bp, 25,174 bp, and 19,504 bp. Geneious v11.0.2 (Kearse et al. 2012) was used to map the three contigs to the reference genome *N. lotus* (NC_041238) to determine the order and their direction (Kim et al. 2019). Gaps and boundaries were extended using MITObim v1.9 (Hahn et al. 2013) with default parameters except for the following: -quick -start 1 -end 250. The chloroplast genome of *N. thermarum* was annotated in DOGMA (Wyman et al. 2004). In addition, visual the genes were inspected manually and curated, and all annotations using Geneious v11.0.2 with *N. lotus* as the reference. The chloroplast genome of *N. thermarum* is publicly available in GenBank under accession number MW143076.

We have obtained a complete circular chloroplast genome with a typical quadripartite structure. The length is 159,849bp, and the GC content is 39.2%. The length of the large single copy (LSC), inverted repeat (IR) and small single copy (SSC) region is 89,997 bp, 25,174 bp, 19,502 bp, and the GC content is 37.8%, 43.4%, and 34.4%, respectively. The plastid genome contains 8 rRNA, 37 tRNA, and 85 protein-coding genes, which is consistent with other water lilies, such as *N. lotus* and *Nymphaea capensis* (NC_040167). The LSC region contains 63 genes, the SSC region contains 12 genes, and the IR region contains five genes.

To determine the phylogenetic position of *N. thermarum* in the *Nymphaea*. 10 Nymphaeaceae chloroplast genomes were selected from GenBank for the phylogenetic analysis with *Barclaya longifolia* and *Nuphar advena* serving as the outgroups. One of the IR repeat regions was removed for the phylogenetic analysis to avoid over-representation of the repeats (Abdullah et al. 2019). The chloroplast genomes were aligned with MAFFT v7.407 (Katoh and Standley 2013) in the automatic mode, and aligning regions were identified and removed using trimAl v1.4 (Capella-Gutiérrez et al. 2009) with the ‘-automated1’ option. Maximum likelihood analysis was performed using IQ-TREE v1.5.1 (Nguyen et al. 2015) with the K3Pu + F + R2 substitution model and 1000 ultrafast bootstrap replicates (Minh et al. 2013). In addition, Bayesian inference (BI) was performed with MrBayes v3.2.6 (Ronquist and Huelsenbeck 2003), using the parameter: lset nst = 6; rates = invgamma; Ngen = 1,000,000; Nruns = 2. The phylogenetic analysis fully resolved a sister relationship for *N. thermarum* and *N. capensis* with a 100% bootstrap value (Figure 1). *Nymphaea ampla*, *N. thermarum* and *N. capensis* classified in subgenera *Brachyceras*, Subg. *Brachyceras* was supported as a sister group to the subgg. *Hydrocallis*-*Lotos* clade with maximum support, consistent with previous analyses (Borsch et al. 2007; Pellicer et al. 2013). This phylogenetic relationship provides reliable evidence for the phylogenetic relationship of the *Nymphaea* genus.

**Figure 1.**
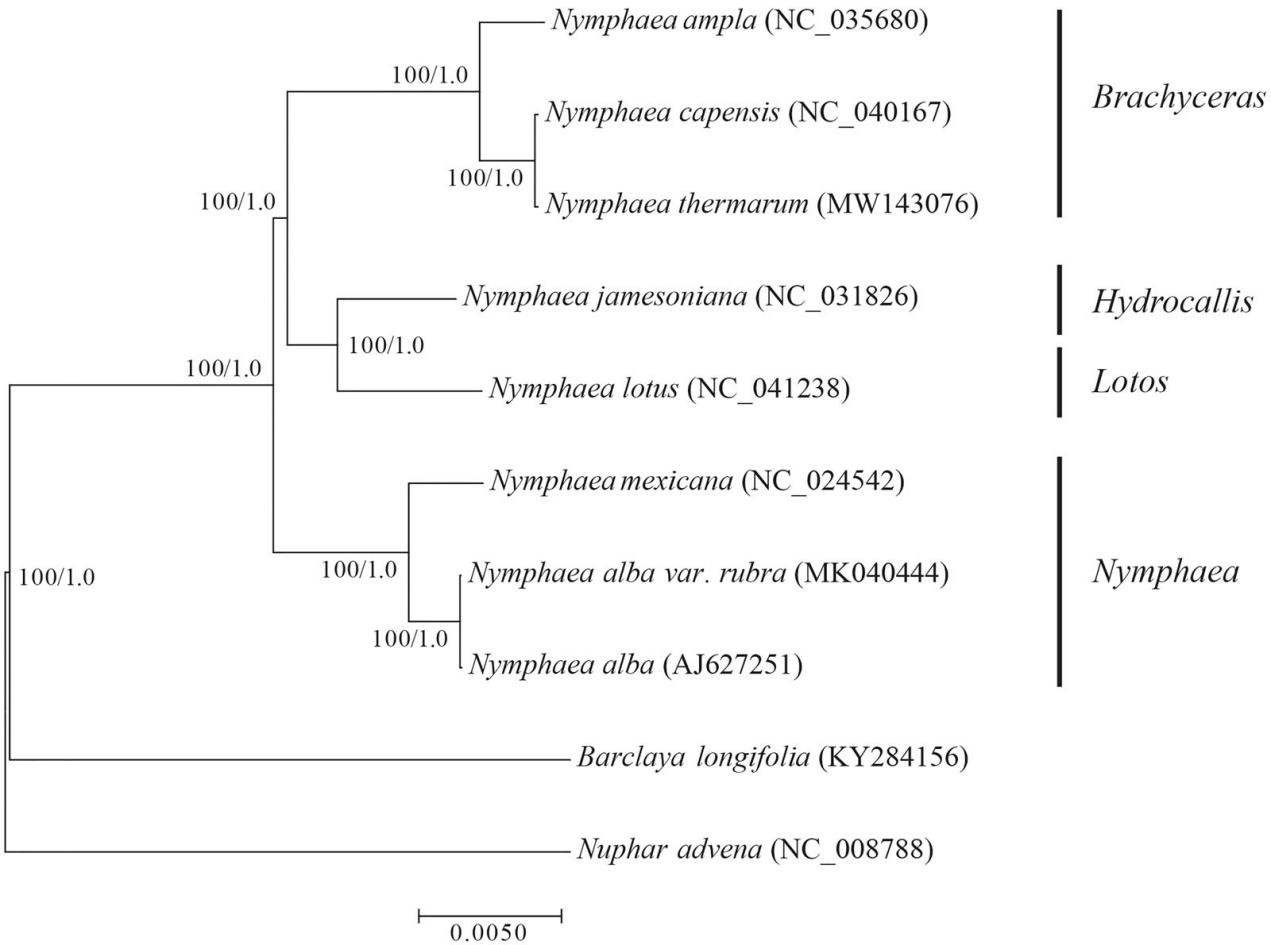
Maximum-likelihood and Bayesian inference phylogeny inferred from 12 Nymphaeales and two outgroup chloroplast genomes. Bootstrap values (left) and posterior probabilities (right) for maximum-likelihood and Bayesian inference analysis are shown on branches with each node.

## Data Availability

The genome sequence data that support the findings of this study are openly available in GenBank of NCBI at (https://www.ncbi.nlm.nih.gov/) under the accession no. MW143076. The associated BioProject, SRA, and Bio-Sample numbers are PRJNA508901, SRR8492137, and SAMN10232690, respectively.
